# Development of dual-functional catalysis for hydrazine oxidation by an organic p–n bilayer through *in situ* formation of a silver co-catalyst†

**DOI:** 10.1039/d1ra07960c

**Published:** 2022-01-12

**Authors:** Mamoru Sato, Toshiyuki Abe

**Affiliations:** Department of Frontier Materials Chemistry, Graduate School of Science and Technology, Hirosaki University 3 Bunkyo-cho Hirosaki 036-8561 Japan tabe@hirosaki-u.ac.jp

## Abstract

Dual-functional catalysis indicates that an organic p–n bilayer induces the catalytic oxidation involved in downhill reactions, not only under illumination but also in the dark. When the organo-bilayer is composed of a perylene derivative (3,4,9,10-perylenetetracarboxylic-bis-benzimidazole (PTCBI), n-type) and cobalt phthalocyanine (CoPc, p-type), only the photocatalytic oxidation of hydrazine (N_2_H_4_) occurs. However, the loading of Ag co-catalyst onto the CoPc surface in the PTCBI/CoPc bilayer successfully led to dual catalysis in terms of the oxidation of N_2_H_4_ to N_2_. To develop the present dual catalysis Ag loading was essential to achieve the catalysis performance particularly without irradiation.

## Abstract 2

An organic p–n bilayer induced the catalysis for hydrazine oxidation both under irradiation and in the dark particularly when Ag particles were employed as co-catalyst.

## Graphical abstract

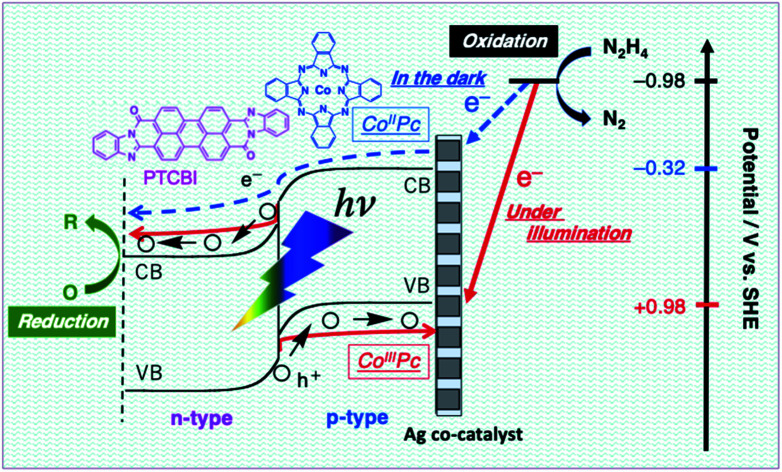

## Introduction

Studies on photocatalytic reactions have been widely reported. In addition to the application in uphill reactions with Δ*G*° > 0 (*e.g.*, water splitting),^1–5^ photocatalysts are also effectively applied in downhill reactions with Δ*G*° < 0 (*e.g.*, decomposition of pollutants), particularly when kinetically severe oxidation with large activation energy is involved.^6–10^ Among the photocatalysts, TiO_2_ is recognized to be practically used in the degradation of several types of pollutant.^11–15^ However, the degrading targets are limited to only substances of low concentrations because of the ultraviolet (UV) response of TiO_2_. Moreover, the catalytic degradation of pollutants by TiO_2_ is expected to occur only under UV irradiation; in other words, TiO_2_ can never exhibit a catalytic performance for degradation in the dark similar to that under irradiation.

Various types of photocatalysts and electrocatalysts towards energy applications are extensively investigated in terms of hydrogen evolution, oxygen evolution, CO_2_ reduction, oxygen reduction, alcohol oxidation, *etc.*, and some reviews have been published recently.^16–20^ We have been studying organic p–n bilayer, including p-type and n-type, semiconductors for application in photoelectrochemical and photocatalytic reactions in the water phase.^21–32^ Some unique reactions, which had not been induced so far by conventional photocatalysts, were also found to occur.^27–32^ In our studies, an organic p–n bilayer using 3,4,9,10-perylenetetracarboxylic-bis-benzimidazole (PTCBI) and cobalt phthalocyanine (CoPc) as n-type and p-type semiconductors, respectively, achieved the catalytic oxidation of thiol under illumination and in the dark.^28^ As depicted in Scheme S1,† the dark reaction occurs according to the pathway indicated with a dashed line, in which the lower edge of the conduction band of CoPc (*i.e.*, Co^II^Pc) corresponds to the potential for oxidizing thiol. While, under irradiation, the thiol oxidation proceeds as demonstrated by the solid-lined pathway, in which the oxidizing power is generated at the CoPc surface (*i.e.*, Co^III^Pc) *via* a series of photophysical events (*i.e.*, formation of excitons based on visible light absorption by the PTCBI/CoPc bilayer, excitation energy transfer of the excitons, generation of carriers by the dissociation of excitons into electrons and holes at the p–n interface, and conduction of hole carriers through the valence band of CoPc). We named “dual-functional catalysis” for the aforementioned catalysis. In each case, electrons can attain the conduction band of PTCBI, followed by its consumption through a reduction reaction. However, such dual-functional catalysis has been achieved only for thiol oxidation.^27,28^

Previously, we reported that the photocatalytic oxidation of hydrazine (N_2_H_4_) occurs at the CoPc surface in the PTCBI/CoPc bilayer,^32^ but there was no evidence of the corresponding oxidation in the dark. In this study, to develop the catalytic oxidation of N_2_H_4_ in the dark, silver species was combined with the organo-bilayer. Ag^33^ and Ag_2_O^34^ are recognized as a catalyst in the dark and photocatalyst, respectively, for N_2_H_4_ oxidation; however, Ag_2_O may be reduced to Ag in the presence of N_2_H_4_ (*i.e.*, reductant). As a result, by preparing the organo-bilayer modified with Ag_2_O, the dual-functional catalysis for N_2_H_4_ oxidation was accomplished. The details are discussed from the perspective of photoelectrochemistry.

## Experimental

PTCBI was synthesized according to a reported procedure and purified by sublimation prior to use.^35^ CoPc was purchased from Tokyo Chemical Industry and used as received. Indium tin oxide (ITO)-coated glass plate (sheet resistance = 8 Ω cm^−2^; transmittance ≥ 85%; and ITO thickness = 174 nm) was acquired from AGC Inc. N_2_H_4_ and Nafion (Nf) alcoholic solution were purchased from Kanto Chemical and Sigma-Aldrich, respectively. All other chemicals employed were of extra pure grade.

The PTCBI/CoPc bilayer was fabricated by vapor deposition (pressure ≤ 1.0 × 10^−3^ Pa; deposition speed = 0.03 nm s^−1^),^32^ in which PTCBI was first coated on an ITO, followed by coating CoPc on top of the PTCBI layer. The thickness of the organo-bilayer was determined by measuring a UV-VIS absorption spectrum. Determination procedure of the thickness with each layer has so far been described elsewhere.^26^ Silver(i) oxide was synthesized according to a reported procedure.^36^ Silver(i) nitrate (0.58 g) was dissolved in water (50 mL), and the pH value of the aforementioned AgNO_3_ solution was adjusted to pH = 14 with 2 M NaOH solution. The resulting solution was maintained by stirring overnight, following which Ag_2_O was collected by filtration and dried at 70 °C. The resulting particles of Ag_2_O (13 mg) were suspended in 1 wt% Nafion (Nf) alcoholic solution (1 mL). The mixture solution (23 μL) was dropped on the CoPc surface in the PTCBI/CoPc bilayer and dried at 70 °C. The fabricated electrode is denoted as PTCBI/CoPc-Nf[Ag_2_O] (*i.e.*, effective area = 1 cm^2^; thickness of Nf = 1 μm; loaded amount of Ag_2_O = *ca.* 1.3 μmol cm^−2^). Nf membrane was employed as the absorbent for N_2_H_4_ and support for Ag_2_O. In the present study, the controlled electrode free of Ag_2_O was also prepared and used (denoted as PTCBI/CoPc-Nf).

The structural analyses of Ag_2_O and Ag were performed using by an X-ray diffractometer (XRD: Rigaku, SmartLab 9 kW), a tunneling electron microscope (TEM: JEOL, JEM-2100) and a scanning electron microscope (SEM: JEOL, JSM-7000F).

When measuring voltammograms and photocurrents for acquiring an action spectrum, a single-compartment cell was operated using a potentiostat (Hokuto Denko, HA-301) equipped with a function generator (Hokuto Denko, HB-104), a coulomb meter (Hokuto Denko, HF-201), and an X–Y recorder (see Scheme S2†). Particularly for the action spectral measurements, the light source was used in the combination with a monochromator (Soma Optics, Ltd, S-10) for irradiating monochromatic light.

**Scheme 1:**
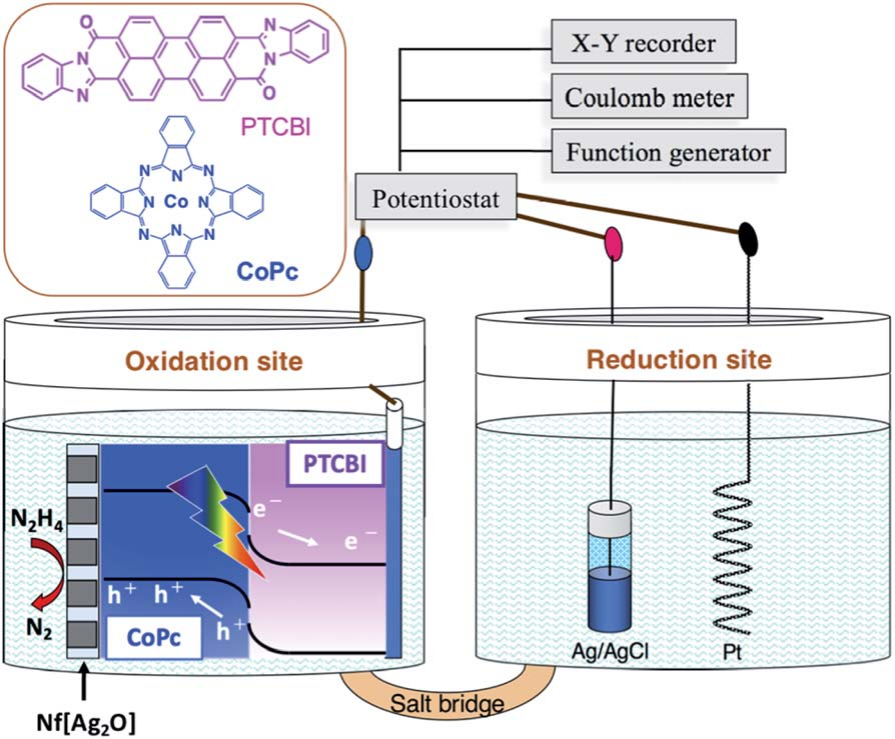
Illustration of the photoelectrolysis system employed for N_2_H_4_ oxidation and chemical structures of PTCBI and CoPc.

The electrolysis study was performed in a twin-compartment cell separated by a salt bridge (Scheme 1). PTCBI/CoPc-Nf[Ag_2_O] and Pt were placed as oxidation site in a N_2_H_4_ solution (5 mM, pH = 11) and as reduction site in a phosphoric acid solution (pH = 0), respectively. Ag/AgCl reference was set along with the Pt counter. For preparing the salt bridge, both agar (1.3 g) and KNO_3_ (4.74 g) were dissolved in hot water (10 mL). Subsequently, the mixture was allowed to flow into the bridging part of the cell, followed by its solidification at room temperature. The twin-compartment cell for electrolysis reaction was operated using the aforementioned electrochemical apparatus.

A halogen lamp was used for irradiating the organo-bilayer. The light intensity was measured using a power meter (type 3A from Ophir Japan, Ltd), and the intensity was determined at approximately 100 mW cm^−2^, except for the action spectral measurement. The gaseous products of N_2_ and H_2_ were analyzed using a gas chromatograph (GL Sciences, GC-3200) equipped with a thermal conductivity detector (column, 5 Å molecular sieve; carrier gas, Ar). Additional experimental details are described in the ESI.†

## Results and discussions

**Fig. 1:**
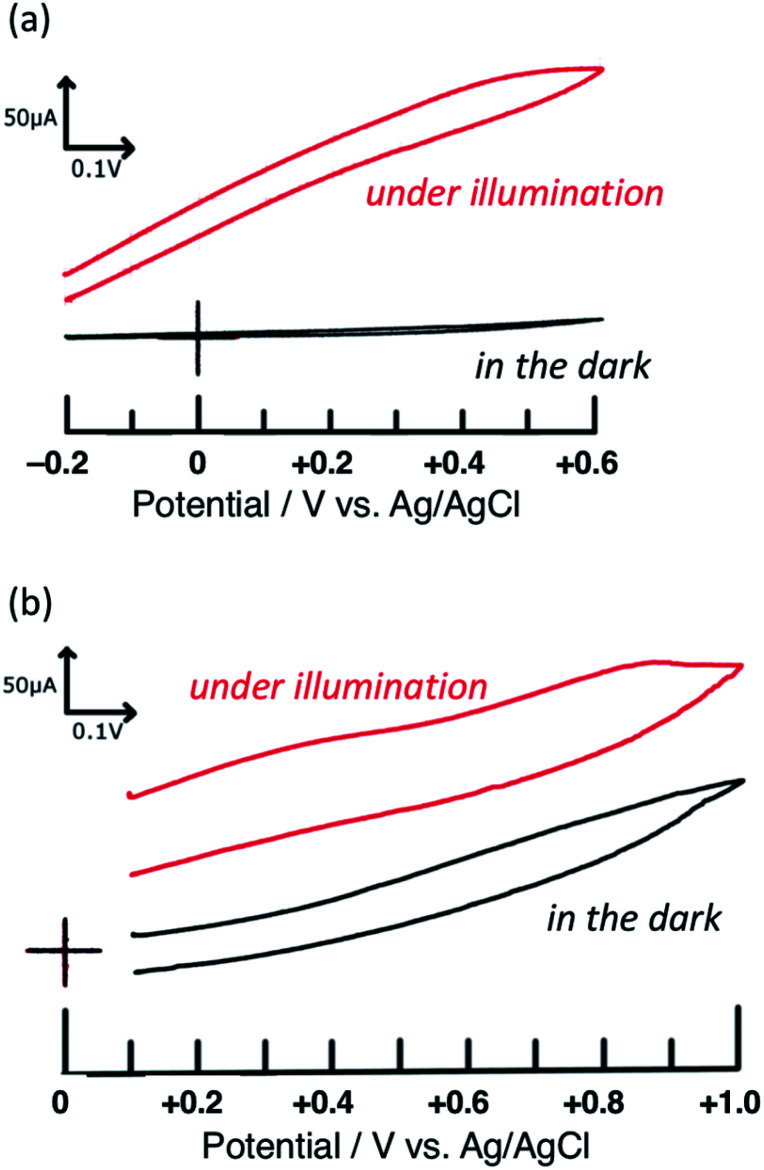
Voltammograms of (a) PTCBI/CoPc-Nf and (b) PTCBI/CoPc-Nf[Ag_2_O]. Film thickness: n-type PTCBI = 230 nm, p-type CoPc = 65 nm, and Nf = 1 μm; N_2_H_4_ solution, 5 mM (pH = 11); light intensity, 100 mW cm^−2^; scan rate = 20 mV s^−1^.

First, the voltammograms of PTCBI/CoPc-Nf[Ag_2_O] were measured in the dark and under irradiation (see Scheme S2†) and compared with those of PTCBI/CoPc-Nf. Similar to our previous study,^32^ PTCBI/CoPc-Nf induced the oxidation of N_2_H_4_ only under irradiation (Fig. 1(a)). However, when loading Ag_2_O on the PTCBI/CoPc bilayer, irrespective of irradiation, anodic currents occurred at PTCBI/CoPc-Nf[Ag_2_O] because of the N_2_H_4_ oxidation (Fig. 1(b)). Electrochemical oxidation of N_2_H_4_ was examined under potentiostatic conditions (see Scheme 1), and the electrolysis data are summarized in Table 1. The oxidative formation of N_2_ from N_2_H_4_ was confirmed in the dark along with the reduction of H^+^ to H_2_. Moreover, the N_2_ (oxidation product) and H_2_ (reduction product) amounts increased significantly under irradiation (note that in each case the faradaic efficiency of the N_2_ and H_2_ formation was estimated to be >85% and >90%, respectively), which are consistent with the aforementioned voltammetric characteristics of Fig. 1(b). As a supplementary explanation, the oxidation of N_2_H_4_ to N_2_H_2_ and subsequent spontaneous decomposition of N_2_H_2_ to N_2_ and H_2_ (ref. 37) are considered not to occur in the present system because no formation of H_2_ was confirmed in the oxidation site in Scheme 1.

**Table 1:** Typical photoelectrolysis data of the decomposition of N_2_H_4_ by PTCBI/CoPc/Nf[Ag_2_O]^a^

	H_2_ evolved/μL	N_2_ evolved/μL	Note
Entry 1	14.3	8.08	In the dark
Entry 2^b^	94.8	48.4	Under irradiation

a Film thickness: PTCBI = 205 nm and CoPc = 60 nm; electrolyte solution (oxidation site), an aqueous N_2_H_4_ solution (5 mM, pH = 11); electrolyte solution (reduction site), an aqueous H_3_PO_4_ solution (pH = 0); applied potential, +0.3 V (*vs.* Ag/AgCl); reaction time, 3 h.

b Irradiation was conducted from the backside of ITO-coated face (light intensity, 100 mW cm^−2^).

**Fig. 2:**
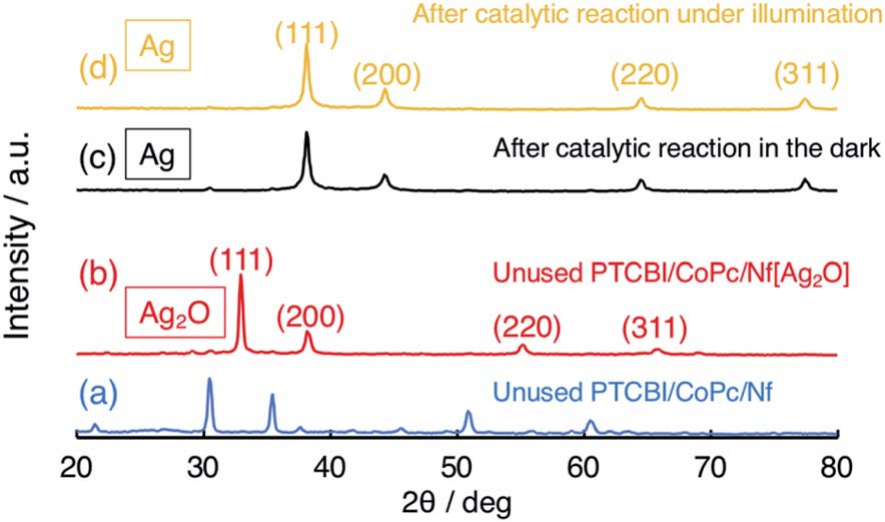
XRD patterns of (a) PTCBI/CoPc-Nf, (b) unused PTCBI/CoPc-Nf[Ag_2_O], and PTCBI/CoPc-Nf[Ag_2_O] after the N_2_H_4_ oxidation (c) in the dark and (d) under irradiation.

It is important to verify how Ag_2_O participated in the N_2_H_4_ oxidation, particularly in the dark (*vide supra*). To clarify the catalytically active Ag species for the N_2_H_4_ oxidation, X-ray diffractometer (XRD) patterns were measured (Fig. 2). For reference, the XRD pattern of PTCBI/CoPc-Nf is shown in Fig. 2(a). In the unused PTCBI/CoPc-Nf[Ag_2_O] (Fig. 2(b)), the resulting XRD pattern was characterized by cubic Ag_2_O.^38^ From the XRD patterns after the electrolysis in the dark (Fig. 2(c)) and under irradiation (Fig. 2(d)), the formation of cubic Ag was confirmed,^39^ indicating a reductive transformation of Ag_2_O in the presence of N_2_H_4_. The XRD pattern depicted in Fig. S1† indicates that the Ag formation is probably occurring during the Ar purge of the electrolyte solution (30 min) prior to the electrochemical measurements. Comparing the potentials of +0.70 V *vs.* SHE (pH = 11) for *E*° (Ag_2_O/Ag)^40^ and −0.98 *vs.* SHE (pH = 11) for *E*° (N_2_H_4_/N_2_),^41^ the reduction of Ag_2_O to Ag can occur reasonably using N_2_H_4_ as reductant (*i.e.*, N_2_H_4_ + 2Ag_2_O → N_2_ + 4Ag + 2H_2_O). Thus, the present dual catalysis for N_2_H_4_ oxidation originates from the *in situ* formation of Ag co-catalyst at the PTCBI/CoPc bilayer, revealing that Ag_2_O does not collaboratively show photocatalytic activity for the N_2_H_4_ oxidation along with the organo-bilayer (*vide supra*). Some TEM and SEM images of Ag_2_O (or Ag) dispersed in Nf membrane were observed. As for the samples prior to electrochemical study, those TEM images indicated the particle sizes were approximately <10 nm and 3–35 nm for Ag_2_O and Ag, respectively (Fig. S2(a) and (b)†). The SEM images were observed for PTCBI/CoPc-Nf[Ag_2_O] exposed to the N_2_H_4_ solution under Ar purge (Fig. S3(a)†) as well as that after photoelectrolysis (Fig. S3(b)†). Each image was almost the same as each other, indicating that Ag transformed from Ag_2_O remains unchanged even after the photoelectrolysis. That is, the aggregation and growth of Ag particles was not be recognized after its use.

**Scheme 2:**
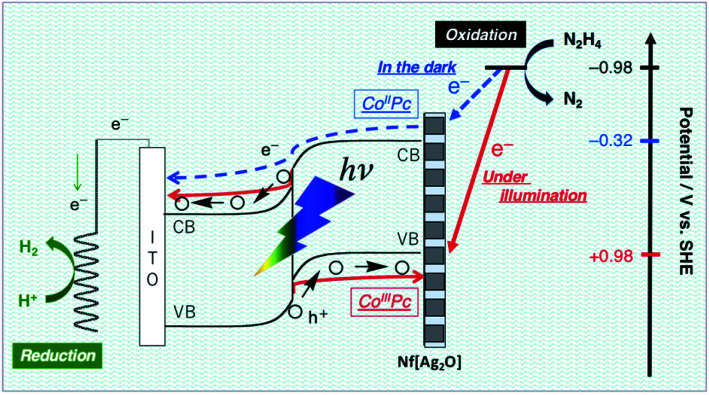
Mechanism of the dual-functional catalysis for N_2_H_4_ oxidation occurring at PTCBI/CoPc-Nf[Ag_2_O] under illumination and in the dark.

**Fig. 3:**
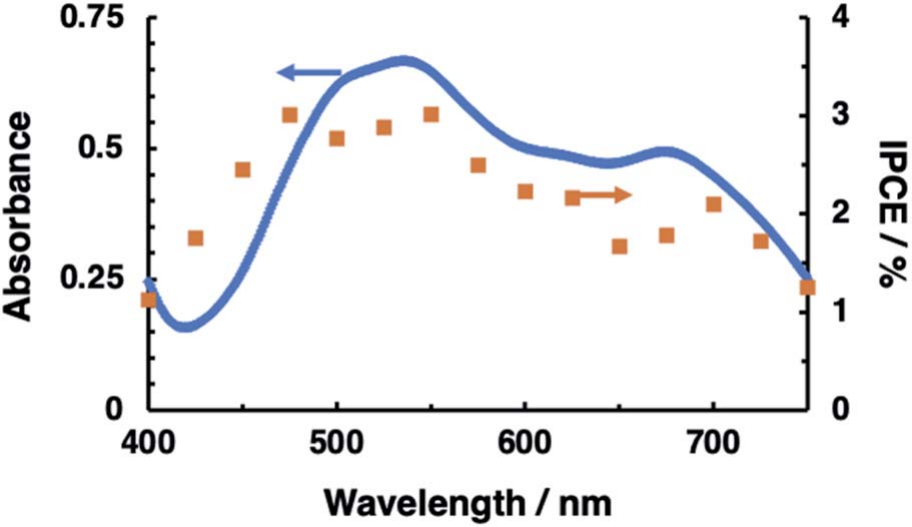
Action spectrum (closed squares) for photocurrents measured at PTCBI/CoPc-Nf (without loading Ag_2_O). The absorption spectrum of PTCBI (solid line) is also depicted. Irradiation was conducted from the backside of ITO-coated face. Film thickness: PTCBI = 210 nm and CoPc = 80 nm; applied potential, +0.3 V *vs.* Ag/AgCl; light intensity, 0.15 mW cm^−2^; electrolyte solution, an aqueous N_2_H_4_ solution (5 mM, pH = 11).

The N_2_H_4_ oxidation occurring at PTCBI/CoPc-Nf[Ag_2_O] is represented in Scheme 2. In the dark, the potential of Co^II^Pc (corresponding to the lower edge of the conduction band: −0.32 V *vs.* SHE, pH = 11)^42^ is available for the N_2_H_4_ oxidation, and thus, N_2_H_4_ is catalytically oxidized at the Ag-loaded CoPc surface. When the photoinduced oxidation of N_2_H_4_ occurs, the oxidizing power is generated at the top edge of the valence band of CoPc (*i.e.*, Co^III^Pc, +0.93 V *vs.* SHE, pH = 11)^42^ through a series of the photophysical events within the organo-bilayer (*vide supra*). According to the resulting action spectrum for photocurrents (Fig. 3), the photoinduced N_2_H_4_ oxidation occurred originating in the absorption of PTCBI over the entire visible light region. This is a specific characteristic usually observed when using PTCBI as the n-type layer.^25,27,28,32^ The oxidizing power is larger under irradiation than in the dark; consequently, the photoinduced N_2_H_4_ oxidation is noticeably superior to the oxidation in the dark. In thermodynamic sense, the N_2_H_4_ oxidation can occur at the CoPc in the dark. However, to induce kinetically the forward oxidation of N_2_H_4_ to N_2_ particularly in the dark, the Ag co-cocatalyst needs to be loaded.

## Conclusion

In summary, the oxidation of N_2_H_4_ to N_2_ occurred successfully at the PTCBI/CoPc bilayer under irradiation and in the dark, particularly by loading Ag on the CoPc surface. The development of the present dual-functional catalysis was attributed to the *in situ* formation of Ag through the reductive transformation of Ag_2_O in the presence of N_2_H_4_ as reductant, whereby the catalytic oxidation of N_2_H_4_ effectively occurred even in the dark. Such catalysis for N_2_H_4_ oxidation did not occur at the Ag-free PTCBI/CoPc bilayer. Therefore, the so-called dual-functional catalysis is a novel catalytic process for oxidation reactions, irrespective of irradiation. The loading of a co-catalyst on an organo-bilayer is expected to expand the application for several types of downhill reactions, opening new opportunities in the field of pollutant degradation.

## Conflicts of interest

There are no conflicts to declare.

## Supplementary Material

RA-012-D1RA07960C-s001
