# Supplementary material for: Development of dual-functional catalysis for hydrazine oxidation by an organic p–n bilayer through in situ formation of a silver co-catalyst
Source: RSC Adv. 2022 Jan 12;12(3):1850–4. doi: 10.1039/d1ra07960c (PMC8979130; doi:10.1039/d1ra07960c)
Supplement: RA-012-D1RA07960C-s001 [file RA-012-D1RA07960C-s001.pdf]

Electronic Supplementary Material (ESI) for RSC Advances.
This journal is © The Royal Society of Chemistry 2022

Supporting Information (SI)

Development of dual-functional catalysis for hydrazine oxidation by organic p-n bilayer through in-situ formation of silver co-catalyst

Mamoru Sato, and Toshiyuki Abe*

Calculation procedures

The **faradaic efficiency (*F.E.*)** was calculated as follows:

i) During the electrolysis reaction, the amount of charge passed was measured using a coulomb meter. The theoretical amount of formed N_2 (or H_2) was calculated from the obtained charge amount.

ii) After conducting the electrolysis reaction, both N_2 and H_2 were quantified by gas chromatography.

Concretely, the *F.E.* value for the formation of N_2 (or H_2) was estimated according to the following equation:

$$
\begin{aligned}
F.E.\ (\%) &= [\text{amount of } N_2 \text{ (or } H_2\text{) evolved}]/[\text{theoretical amount of } N_2 \text{ (or } H_2\text{)}] \times 100 \\
&= [\text{amount of } N_2 \text{ (or } H_2\text{) evolved}]/[(\text{amount of charge passed})/(nF)] \times 100
\end{aligned}
$$

where n is the number of electrons participating in the evolution of the products (n = 4 and 2 for the formation of N_2 and H_2, respectively) and F represents the Faraday's constant (9.65×10^4 C mol^{-1}).

The **incident photon-to-current conversion efficiency (*IPCE*)** was estimated according to the following equation:

$$IPCE\ (\%) = ([I/e]/[W/\varepsilon]) \times 100$$

where I (A cm^{-2}) is the photocurrent density, e (C) denotes the elementary electric charge, W (W cm^{-2}) is the light intensity, and ε is the photon energy of monochromatic light.

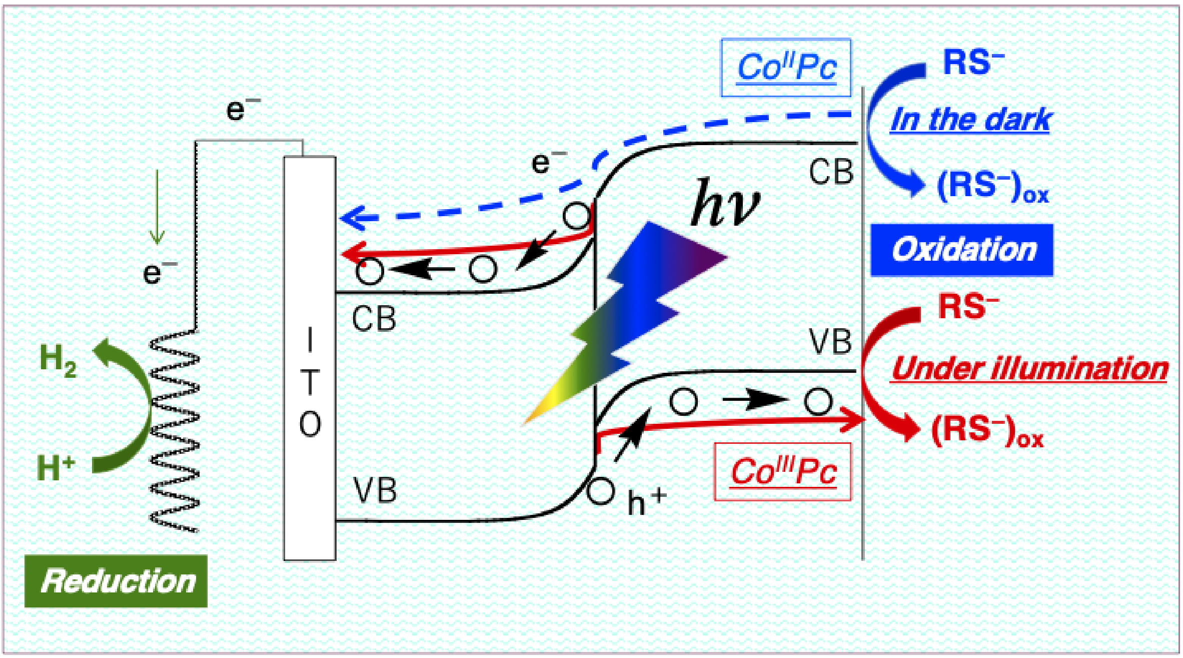

Scheme S1 Mechanism of the dual-functional catalysis for thiol (RS^{-}) oxidation occurring at PTCBI/CoPc-Nf in the dark and under illumination.

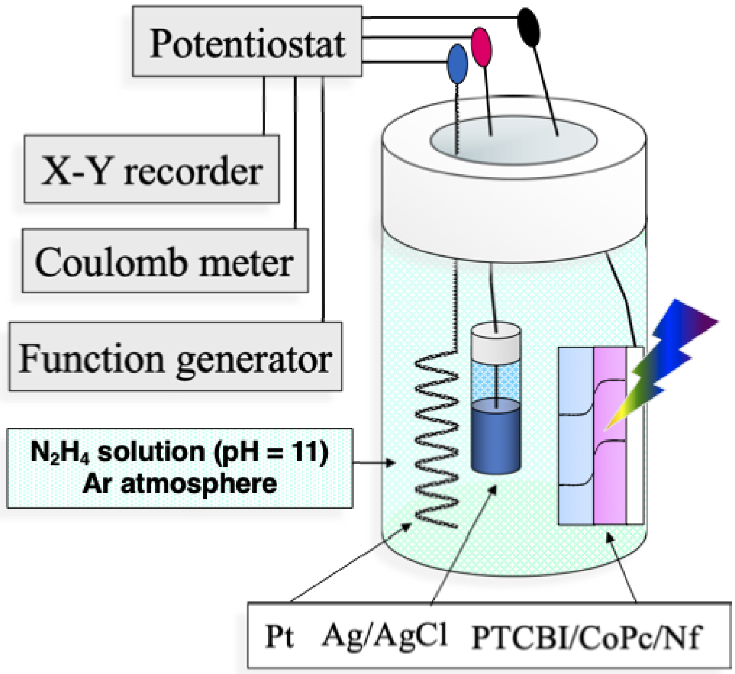

Scheme S2 Illustration of the photoelectrochemical cell employed for measuring both voltammograms and photocurrents under potentiostatic conditions.

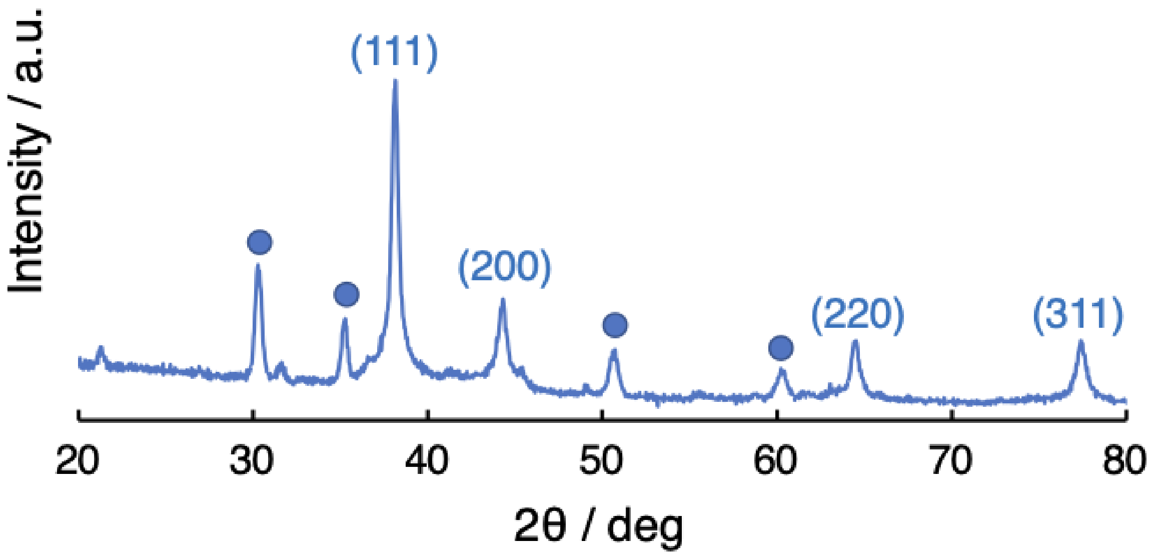

Fig. S1 XRD pattern of PTCBI/CoPc-Nf[Ag_2O] measured after Ar purge of the N_2H_4 solution for 30 min. As for the closed circles, those are consistent with Fig. 2(a).

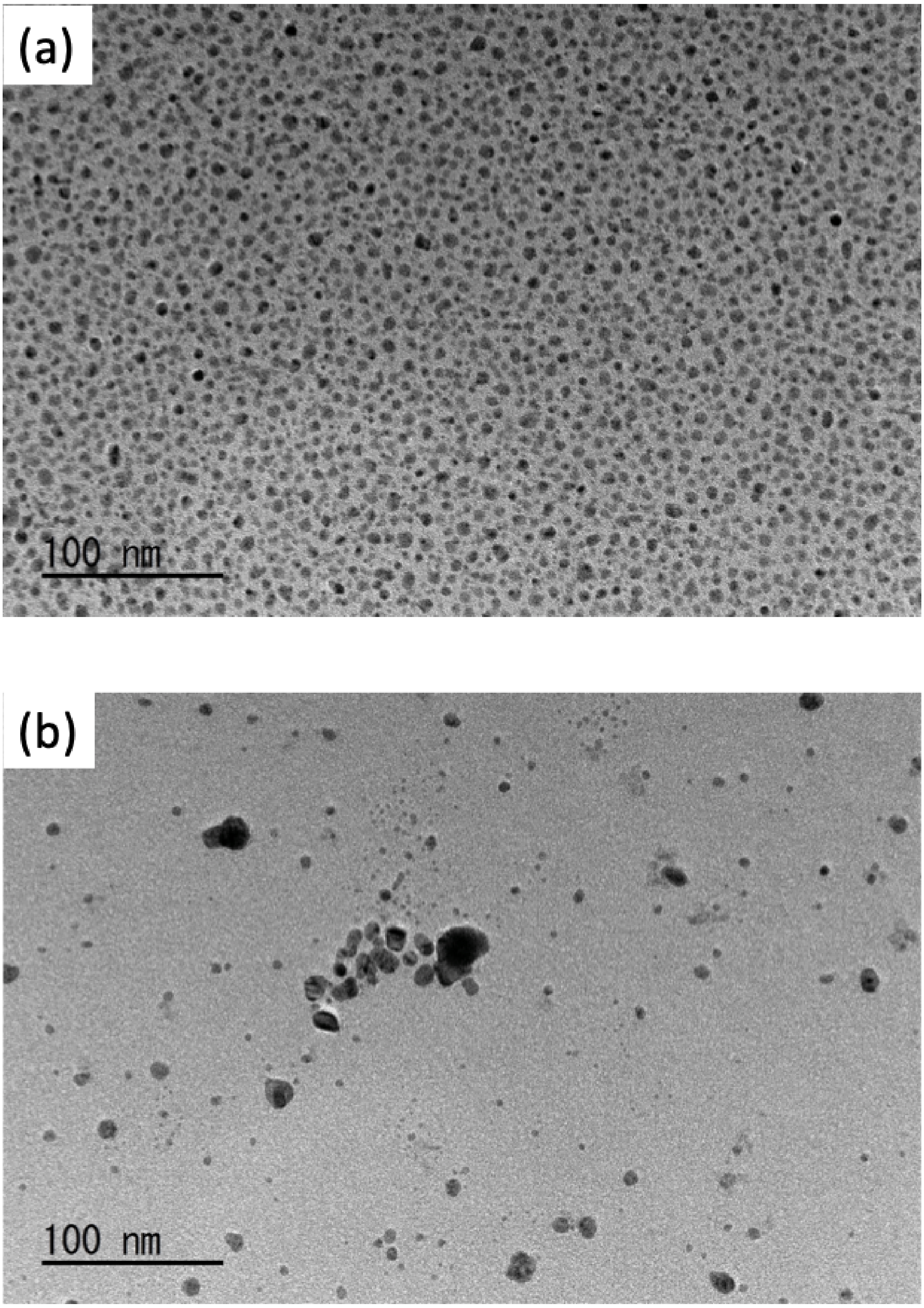

Fig. S2 TEM images of Ag_2O dispersed in a Nf membrane. A 4×10^{-5} dm^3 portion of the suspension, composed of Nf and Ag_2O (see main text), was cast onto a collodion membrane supported on a copper grid mesh and dried at room temperature.

(a), Ag_2O sample after drying; (b), the sample exposed to a N_2H_4 solution under Ar purge (i.e., Ag).

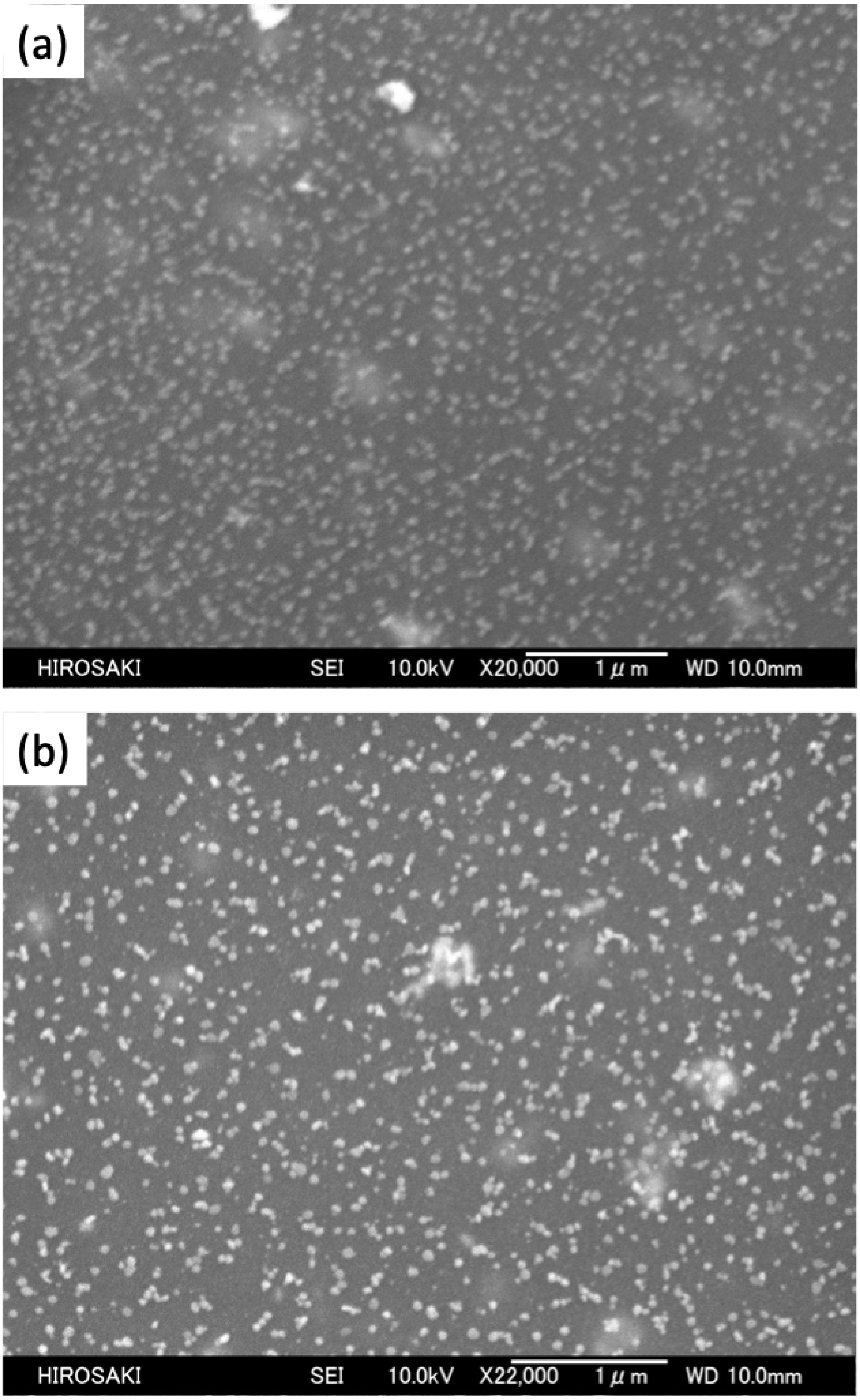

Fig. S3 SEM images of PTCBI/CoPc-Nf[Ag_2O].

(a), the sample exposed to a N_2H_4 solution under Ar purge (i.e., Ag); (b), the sample after the photoelectrolysis.
